# Soluble epoxide hydrolase inhibition decreases reperfusion injury after focal cerebral ischemia

**DOI:** 10.1038/s41598-018-23504-1

**Published:** 2018-03-27

**Authors:** Ranran Tu, Jillian Armstrong, Kin Sing Stephen Lee, Bruce D. Hammock, Adam Sapirstein, Raymond C. Koehler

**Affiliations:** 1 Department of Neurology, Second Xiangya Hospital, Central South University, Changsha, China; 2 Department of Anesthesiology and Critical Care Medicine, Johns Hopkins University, Baltimore, MD USA; 3 Department of Entomology and Nematology and UCD Comprehensive Cancer Center, University of California, Davis, CA USA

**Keywords:** Cell death in the nervous system, Neuro-vascular interactions, Translational research, Stroke

## Abstract

Epoxyeicosatrienoic acids (EETs) are produced by cytochrome P450 epoxygenases from arachidonic acid, and their rapid metabolism is mainly through soluble epoxide hydrolase (sEH). EETs exert vasodilatory, anti-inflammatory, anti-apoptotic, and pro-angiogenic effects. Administration of sEH inhibitors before or at the onset of stroke is protective, but the effects of post-treatment at reperfusion, when inflammation is augmented, has not been as well studied. We tested the hypothesis that 1-Trifluoromethoxyphenyl-3-(1-propionylpiperidin-4-yl)urea (TPPU), a potent and highly selective sEH inhibitor, suppresses inflammation and protects the brain when administered at reperfusion. Vehicle or 1 mg/kg TPPU was administered at reperfusion after 90 minutes of focal ischemia and again 24 hours later. Protein expression and activity of sEH increased after reperfusion and activity was decreased by TPPU administration. TPPU decreased infarct volume by 50%, reduced neurologic deficits and improved performance on sensorimotor tasks. Furthermore, TPPU significantly lowered the mRNA expression of interleukin-1beta by 3.5-fold and tumor necrosis factor-alpha by 2.2-fold, increased transforming growth factor-beta mRNA by 1.8-fold, and augmented immunostaining of vascular endothelial growth factor in peri-infarct cortex. Thus, inhibition of sEH at reperfusion significantly reduces infarction and improves sensorimotor function, possibly by suppressing early proinflammatory cytokines and promoting reparative cytokines and growth factors.

## Introduction

Thrombolysis with tissue plasminogen activator and endovascular thrombectomy are currently the major treatments for patients with acute ischemic stroke. However, for some patients, reperfusion after thrombolysis and thrombectomy could exacerbate the injury by triggering multiple pathologic processes, including the inflammatory cascade, lipid peroxidation, mitochondrial dysfunction, and disruption of the blood-brain barrier. Therefore, identification of agents that target multiple mechanisms during reperfusion would be beneficial for limiting progressive neuronal cell death and promoting an environment that facilitates brain repair.

Epoxyeicosatrienoic acids (EETs) are lipid metabolites produced from arachidonic acid by cytochrome P450 (CYP) epoxygenases. In brain, EETs are predominantly generated by subfamilies of CYP2C and CYP2J, which are expressed in astrocytes as well as vascular endothelium^1–5^. In various organs, EETs exert broadly protective effects, including anti-apoptotic, anti-inflammatory, vasodilatory, anti-nociceptive, anti-epileptic, and pro-angiogenic effects^6–14^. However, EETs are rapidly hydrolyzed by soluble epoxide hydrolase (sEH) into less bioactive 1,2-diols, dihydroxyeicosatrienoic acids (DHETs)^15^. The sEH enzyme is broadly distributed throughout the central nervous system, with cellular expression in astrocytes, neurons, and vascular endothelium^16,17^. Gene deletion of sEH has been shown to reduce infarct volume after transient middle cerebral artery occlusion (MCAO) in male mice^8,18,19^ and reproductively senescent female mice^20^. However, sEH male knockout show a smaller decrease in cerebral blood flow during MCAO, thereby making it difficult to discern direct neuronal protection from effects of a less severe insult. Inhibitors of sEH have also been shown to reduce stroke infarct volume in mice and rats, stroke-prone spontaneously hypertensive male rats, and diabetic male mice^8,21–24^, but the inhibitors were administered as a pretreatment or at the onset of MCAO. With the success of endovascular thrombectomy in establishing recanalization and reperfusion in selected subpopulations of stroke patients, use of neuroprotective agents at the time of reperfusion is of clinical relevance. With regard to sEH inhibitors, the few studies of treatment at reperfusion have focused only on infarct volume as an endpoint^8,20^. Neurobehavior testing was not reported in these studies. Thus, limited data exists on the effect of sEH inhibitor administration at reperfusion and its effects on reperfusion injury.

Neuroinflammation is an important component of reperfusion injury. Early release of proinflammatory cytokines is generally believed to contribute to the spread of infarction, whereas delayed release of anti-inflammatory cytokines contributes to the resolution of the infarction and the initiation of reparative mechanisms^25^. EETs have been recognized as possessing anti-inflammatory properties in a variety of settings, such as lipopolysaccharide-induced inflammation^26,27^ and neuropathic pain^28^. However, under the conditions of cerebral ischemia, the effect of sEH inhibitors on cerebral cytokine expression is not clear-cut. Administration of an sEH inhibitor before MCAO or at reperfusion failed to attenuate cerebral induction of several proinflammatory cytokines^29^, whereas continuous intraventricular infusion of an inhibitor attenuated expression of inducible nitric oxide synthase^23^. In a model of cardiac arrest, administration of an sEH inhibitor after resuscitation failed to attenuate expression of proinflammatory interleukin-1β (IL-1β) or tumor necrosis factor-α (TNF-α)^30^.

The main objectives of the present study were to better characterize the response to administration of an sEH inhibitor at the time of reperfusion by examining effects on tissue cytokine responses, microglia number, and neurobehavior, in addition to infarct volume. Moreover, we used the sEH inhibitor 1-(1-propanoylpiperidin-4-yl)−3-[4-(trifluoromethoxy)phenyl]urea (TPPU). This newer generation sEH inhibitor possesses higher potency and a longer circulatory half-life^31–33^ than many of the inhibitors previously used in stroke models. It also is taken up in rodent brain^34^. We tested the hypothesis that systemic administration of TPPU starting at reperfusion after MCAO in male rats reduces infarct volume, improves sensorimotor functional outcome, suppresses expression of proinflammatory IL-1β and TNF-α, augments expression of anti-inflammatory IL-10 and transforming growth factor-β (TGF-β), and decreases the number of peri-infarct microglia.

## Results

### Localization and expression of sEH in rat brain

**Figure 1:**
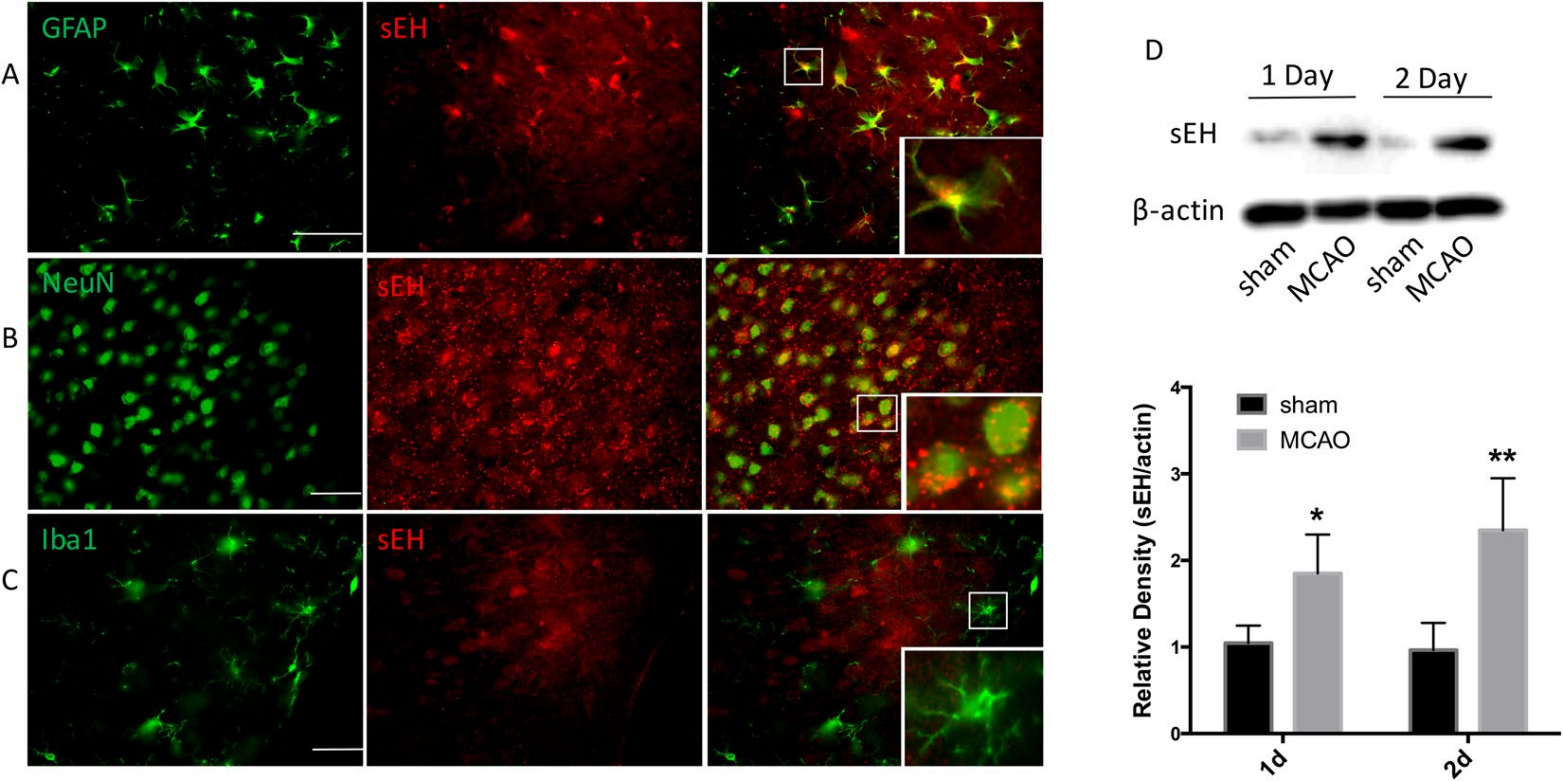
Localization of sEH in rat brain and its expression after ischemia. Representative images show double immunofluorescent staining of sEH (red), GFAP (**A**, green), NeuN (**B**, green), and Iba1 (**C**, green) in cerebral cortex under normal conditions. Scale bar = 50 μm for low power images. Area in white square is shown in inset at higher power. (**D**,**E**) Western blot of sEH expression on 1 and 2 days after reperfusion in the area around the ischemic lesion. The sEH and β-actin bands were stripped and cropped from the same gel and were developed with the same exposure. Images of the full gel are shown in Supplementary Figure S1. Comparisons between sham and MCAO groups were made by *t*-test (means ± SD; *n* = 5 rats per group; *p < 0.05, **p < 0.01 vs sham group).

Localization of sEH has been described in mouse brain^17^, but less information is available in rat cerebral cortex^16^. In non-ischemic rat cortex, sEH immunoreactivity was observed to be widely distributed (Fig. 1A–C). Double-labeling immunofluorescence indicated that sEH co-localized with both glial fibrillary acidic protein (GFAP)-positive astrocytes (Fig. 1A) and NeuN-positive neurons (Fig. 1B), but rarely with ionized calcium-binding adapter molecule 1 (Iba1)-positive microglia (Fig. 1C). Moreover, the morphologic features of the cells suggested that the expression of sEH was largely confined to astrocytes.

Subsequently, we investigated the temporal changes of sEH expression after ischemic stroke. Western blotting showed that the protein expression of sEH significantly increased 1 and 2 days after MCAO compared with that of the sham control (*p* < 0.05; *n* = 5 rats per group; Fig. 1D).

### TPPU inhibits brain sEH activity

**Figure 2:**
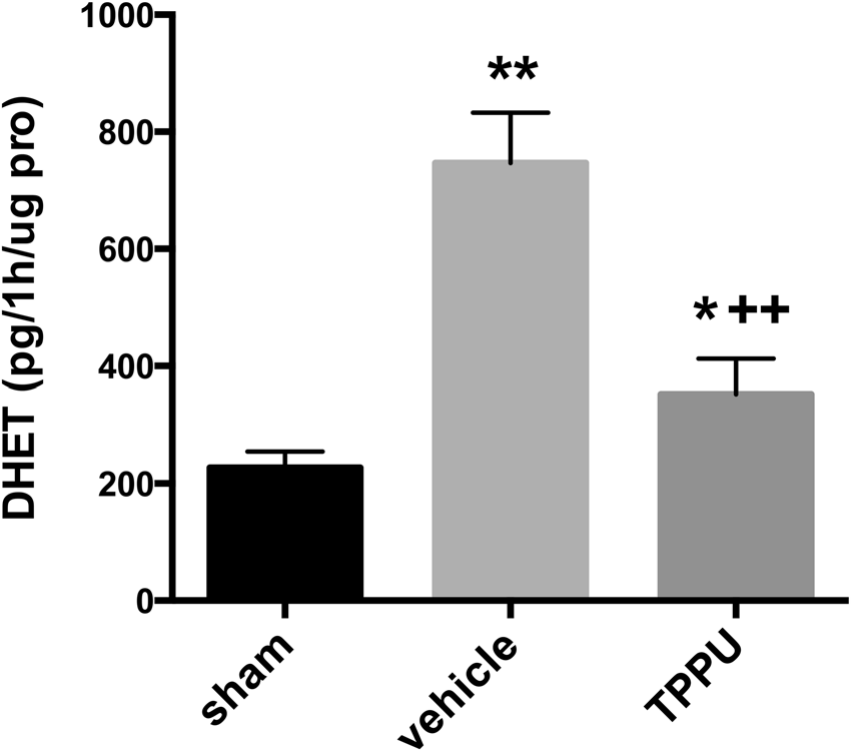
TPPU suppresses sEH activity. The 14,15-DHET production was measured as pg per h per µg of protein (pro) by ELISA 1d after MCAO. Sham, MCAO with vehicle, and MCAO with TPPU groups were compared by ANOVA and the Holm-Sidak procedure for multiple comparisons (means ± SD; *n* = 4 rats per group; *p < 0.05 vs. sham group; **p < 0.01 vs. sham group; ^++^*p* < 0.01 vs. vehicle group).

We then assessed the enzymatic activity of sEH activity in brain parenchyma by measuring the rate of conversion of 14,15-EET to 14,15-DHET with ELISA. Compared with that of the sham group, sEH activity significantly increased 1 day after MCAO in the vehicle group (Fig. 2), indicating that the increase in protein expression after reperfusion was associated with increased activity. This increased activity after MCAO was suppressed in the TPPU-treated group (*p* < 0.01; *n* = 4 rats per group), although activity remained slightly elevated relative to the sham value. Thus, TPPU produced the intended effect of limiting sEH activity.

### TPPU decreases infarct volume

**Figure 3:**
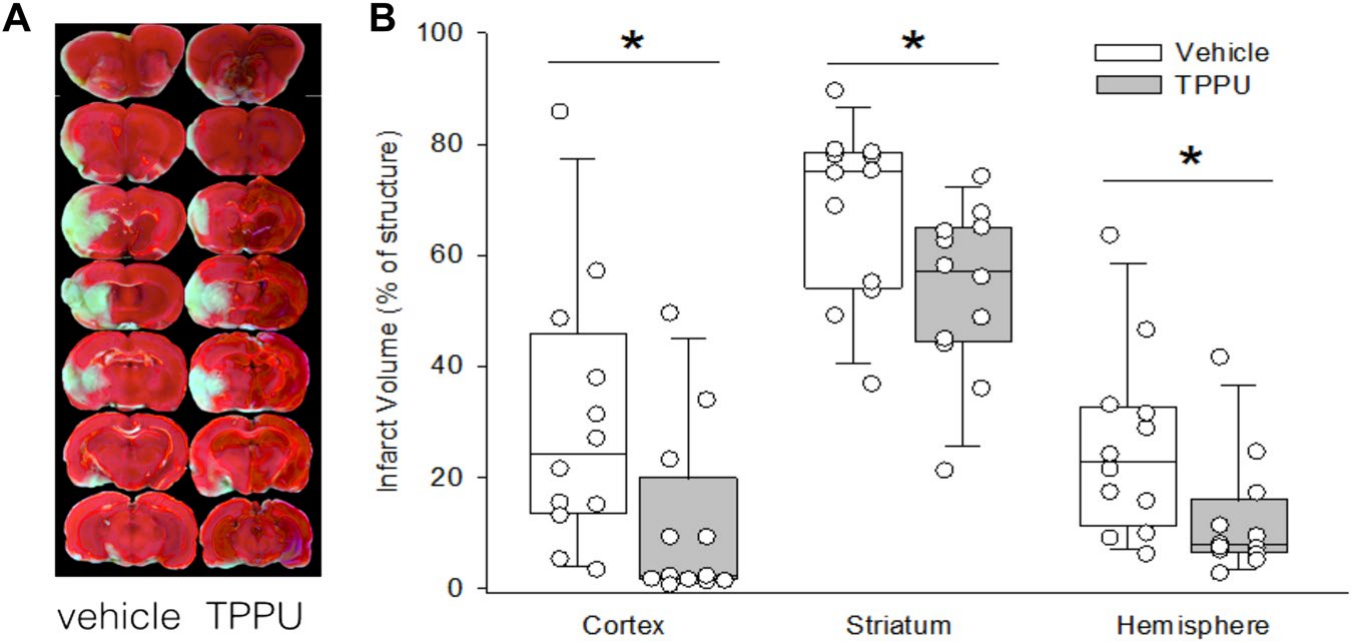
TPPU decreases infarct volume. Representative images of TTC-stained brain sections show that infarct volume was smaller in rats administered TPPU at reperfusion than in those administered vehicle. Individual values of infarct size percentage in cortex, striatum, and hemisphere obtained at 2 d are shown along with box-whisker plots of the median and the 5, 25, 75, and 95% ranges. Comparisons between the vehicle and TPPU groups were made with the Mann-Whitney test (*n* = 12 rats per group; *p < 0.05 vs. vehicle group).

Rats were subjected to 90 min of MCAO, and then 1 mg/kg TPPU or vehicle was administered at reperfusion and again 24 h later. Infarct volume was measured at 48 h after reperfusion. Statistical comparisons between the two groups were made by Mann-Whitney test (*n* = 12 rats per group). In the vehicle group, the median and interquartile range (IQR) of infarct volume in cortex, striatum, and hemisphere were 24% (IQR, 14–46%), 75% (IQR, 54–78%), and 23% (IQR, 11–33%), respectively (Fig. 3). In the TPPU group, the corresponding values were 2% (IQR, 1–20%; *p* = 0.012), 57% (IQR, 44–65%; *p* = 0.023), and 8% (IQR, 6–16%; *p* = 0.023). Thus, post-treatment with TPPU markedly decreased infarct volume.

When pooling rats with vehicle treatment from these and other outcome measurement cohorts, nine rats died within 24 h after reperfusion, and three died between 24 and 48 h, resulting in a mortality rate of 16.7% (12/72). With TPPU treatment, in contrast, only two rats died within 24 h, and one died between 24 and 48 h after reperfusion, resulting in a mortality rate of 4.8% (3/63). This trend of mortality rate was not statistically different (*p* = 0.055, Chi-squared test). The lack of a greater mortality after TPPU treatment indicates that the smaller infarct volume seen with TPPU was unlikely due to greater attrition of rats with worse injury.

### TPPU improves neurobehavior

**Figure 4:**
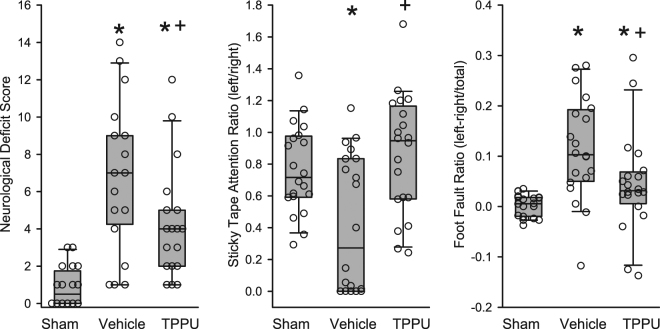
TPPU improves neurologic function at 2 days after MCAO. After right MCAO, (**A**) the neurologic deficit score was less in the TPPU group than in the vehicle group, (**B**) TPPU restored the time ratio of the contralateral left forelimb attention/right forelimb attention in the modified sticky tape test, and (**C**) TPPU decreased the foot-fault index ([left limb faults − right limb faults]/total foot faults). Individual data are shown along with box-whisker plots of the median and the 5, 25, 75, and 95% ranges. The Kruskal-Wallis analysis of ranks test indicated significant treatment effects for all measurements and pairwise comparisons were made with the Mann-Whitney test (*n* = 20 rats per group; *p < 0.05 vs. sham; ^+^*p* < 0.05 vs. vehicle).

At 2 days of recovery, we performed a neurologic assessment as described in Supplementary Table S1. Rats in the vehicle group exhibited a wide range of neurologic deficit scores that were significantly worse than those of a sham-operated group (Fig. 4A). The deficit score was attenuated in the TPPU-treated group (*p* = 0.023; *n* = 20 rats per group). In addition, the modified sticky tape test was used to assess sensory deficits. Attention to the affected forelimb relative to the unaffected limb was decreased in the vehicle group compared to that in the sham group (Fig. 4B). This ratio of attention to the two sides was significantly improved in the TPPU group (*p* = 0.003; *n* = 20 rats per group) and not significantly different from that in the sham group. We also performed a foot-fault test during walking on a grid. The difference between the contralateral and ipsilateral foot faults was greater in the vehicle group than in the sham group (Fig. 4C), but this increase was attenuated in the TPPU group (*p* = 0.014; *n* = 20 rats per group).

### TPPU inhibits neuronal death in the peri-ischemic area

**Figure 5:**
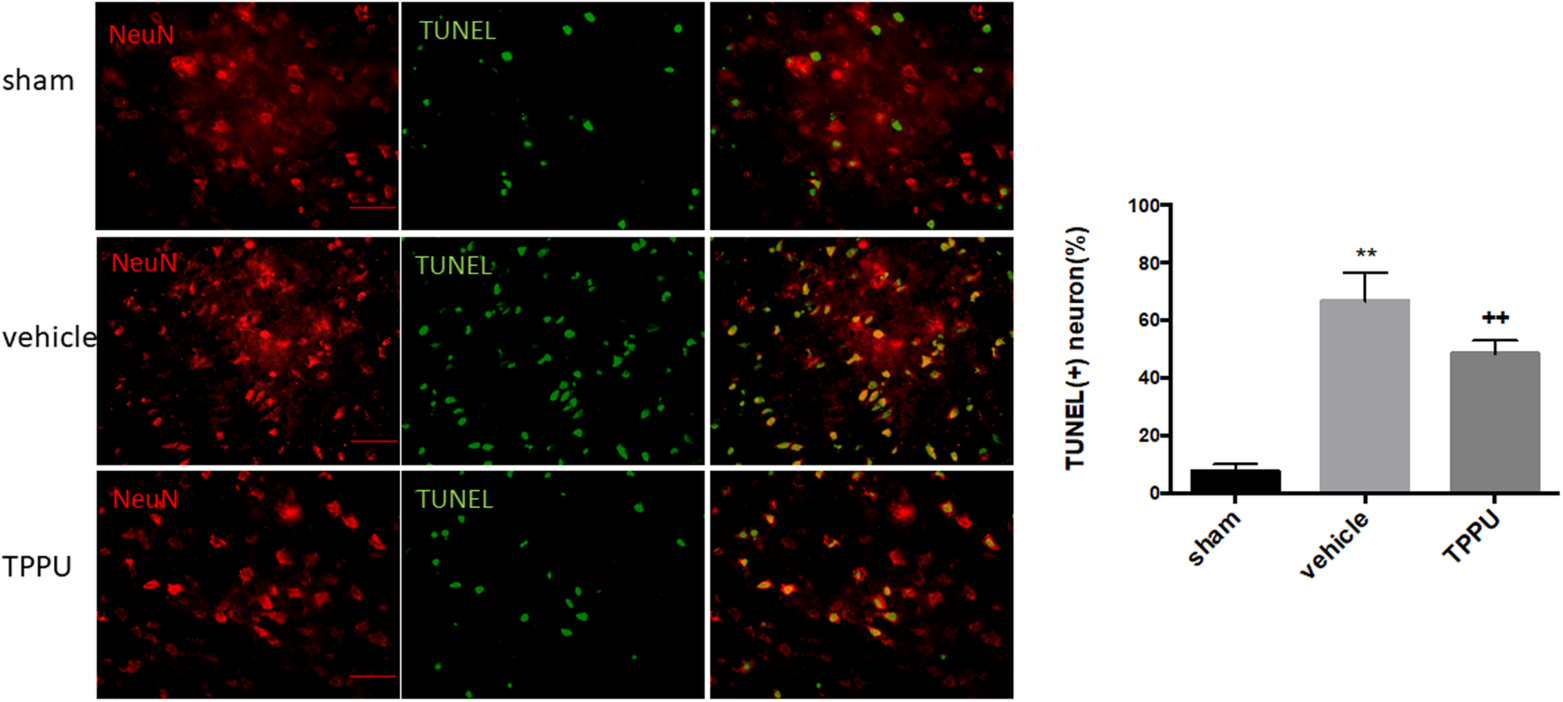
TPPU decreases peri-infarct neuronal cell death. Neuronal cell death in the ipsilateral cortex at 2 d after surgery was detected by terminal deoxynucleotidyl transferase dUTP nick end labeling (TUNEL, green) and NeuN (red) double staining. Scale bar = 50 μm. Sham, MCAO with vehicle, and MCAO with TPPU groups were compared by ANOVA and the Holm-Sidak procedure for multiple comparisons (means ± SD; *n* = 5 rats per group; **p < 0.01 vs. sham group, ^++^*p* < 0.01 vs. vehicle group).

To assess the effects of TPPU on neuronal cell death in the peri-infarct areas of cortex, we double stained with terminal deoxynucleotidyl transferase mediated dUTP nick end labeling (TUNEL) and the neuronal marker NeuN. Staining revealed an obvious increase in the TUNEL-positive neurons at day 2 after the ischemic insult, in contrast with the sparse distribution in sham groups (Fig. 5). TPPU mitigated neuronal cell death in the peri-infarct region by 18% compared with that in the vehicle group (*p* < 0.01; *n* = 5 rats per group).

### TPPU inhibits expression of proinflammatory cytokines

**Figure 6:**
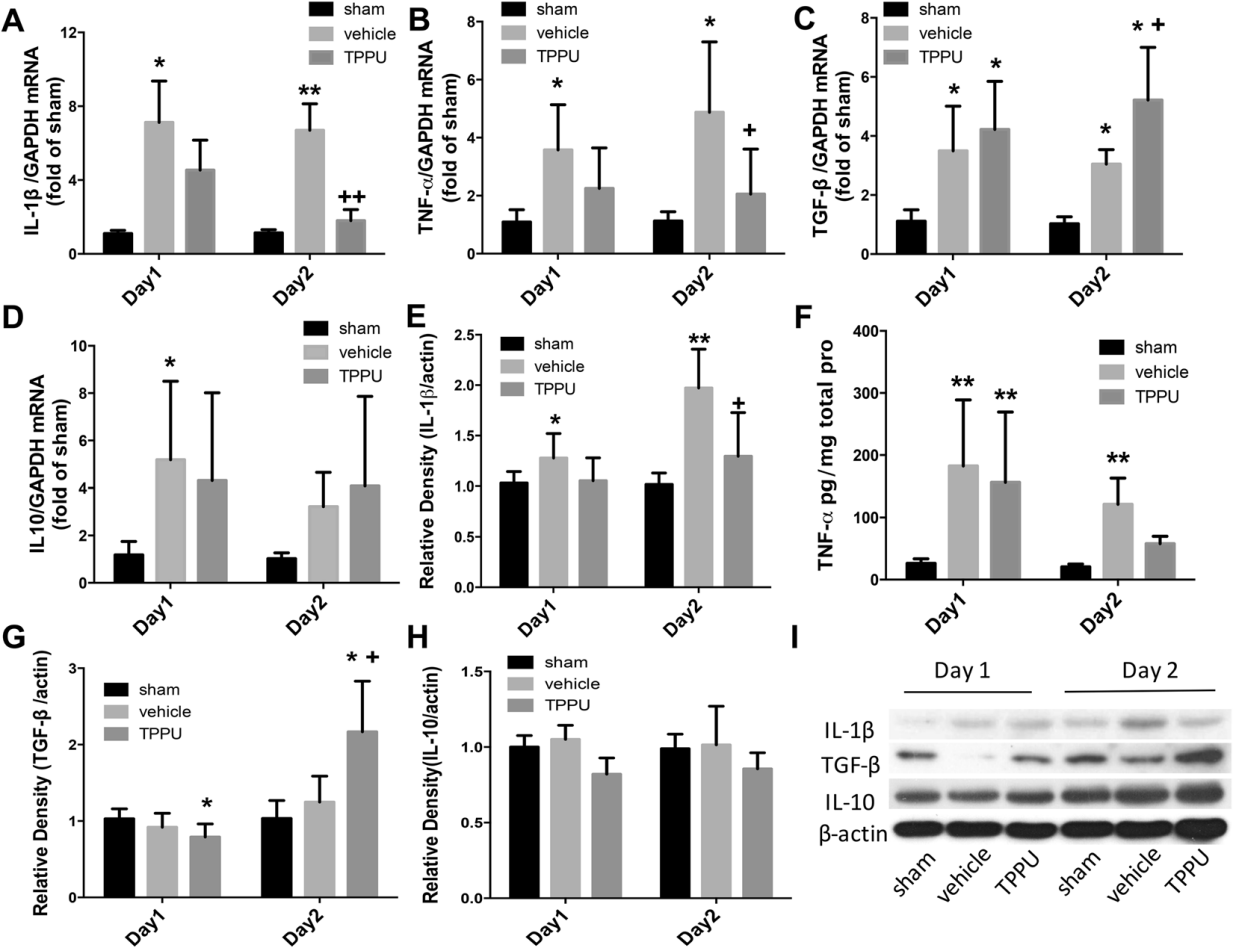
TPPU modifies inflammatory cytokine expression at 1 and 2 d after surgery. (**A**–**D**) Real-time PCR was used to analyze mRNA expression of inflammatory factors. Logarithmically transformed data were subjected to ANOVA and the Holm-Sidak procedure (means ± SD). (**E**–**G**, **I**) Protein levels of IL-1β, IL-10, and TNF-β were measured by Western blot. IL-1β, TGF-β and β-actin bands were stripped and cropped from the same gel and were developed with the same exposure. To avoid overlapping bands, IL-10 was run on a separate gel from the same sample. Images of the full gels are shown in Supplementary Figure S2. (**H**) TNF-α expression was analyzed by ELISA. Data were analyzed with Kruskal-Wallis analysis of ranks, which, when significant, was followed by the Mann-Whitney test for pairwise comparisons. For (**A**–**H)**, *n* = 6 rats per group; *p < 0.05, **p < 0.01 vs. sham group; ^+^*p* < 0.05, ^++^*p* < 0.01 vs. vehicle group.

Inflammatory cytokines play a major part in the development of secondary injury after reperfusion, mediating the recruitment of inflammatory cells to the ischemic lesion. We determined the expression of proinflammatory cytokines IL-1β and TNF-α and anti-inflammatory factors IL-10 and TGF-β by both real-time PCR and Western blot analysis or ELISA. As shown in Fig. 6A–D, expression levels of IL-1β and TNF-α mRNA were elevated at days 1 and 2 after stroke in the vehicle group, but TPPU treatment significantly attenuated expression at day 2 (*p* < 0.05; *n* = 6 rats per group). Expression of TGF-β mRNA also was elevated on days 1 and 2 after MCAO in the vehicle group and was increased even further on day 2 in the TPPU group (*p* < 0.05 vs. vehicle group). However, IL-10 mRNA did not differ significantly between the vehicle and TPPU groups.

Using Western blot analysis and ELISA, we obtained results that generally paralleled the mRNA results (Fig. 6E–I). Protein levels of IL-1β and TNF-α increased on days 1 and 2 in the vehicle group, and TPPU significantly attenuated the increase in IL-1β (*n* = 6 rats per group). With TPPU treatment, TNF-α remained significantly elevated relative to the sham group on day 1, but was no longer significantly different from the sham level on day 2. TPPU treatment significantly increased TGF-β on day 2 relative to both the sham and vehicle groups (*p* < 0.002; *n* = 6 rats per group). No significant changes in IL-10 protein were detected in either MCAO group relative to the sham group. Thus, TPPU treatment generally limited expression of proinflammatory cytokines and augmented expression of at least one of the anti-inflammatory cytokines.

### TPPU inhibits activation of microglia

**Figure 7:**
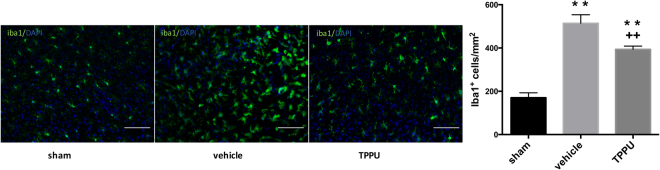
TPPU reduces microglia number in peri-ischemic region. Representative images of Iba1 staining in the peri-ischemic cortex in sham, vehicle, and TPPU-treated groups at day 2 after surgery. Note that enlarged cell bodies in the peri-infarct region of the vehicle group were less prominent in the TPPU group. Scale bar = 50 μm. The number of Iba1-positive cells was compared among groups by ANOVA and the Holm-Sidak procedure for multiple comparisons (means ± SD; *n* = 5 rats per group; **p < 0.01 vs. sham group; ^++^*p* < 0.01 vs. vehicle group).

We evaluated the number and morphologic characteristics of microglia after ischemia by immunolabeling them with Iba1. In the sham group, microglia were typically resting cells with small cell bodies and long, ramified processes (Fig. 7). At day 2 after ischemia, the number of Iba1-positive cells around the ischemic lesion was elevated, and the activated microglia exhibited the expected hypertrophic cell bodies with short processes. In comparison to the vehicle-treated group, the TPPU-treated group had 23% fewer microglia (*n* = 5 rats per group), which exhibited relatively less cell body hypertrophy.

### TPPU increases neuronal and astrocyte VEGF expression

**Figure 8:**
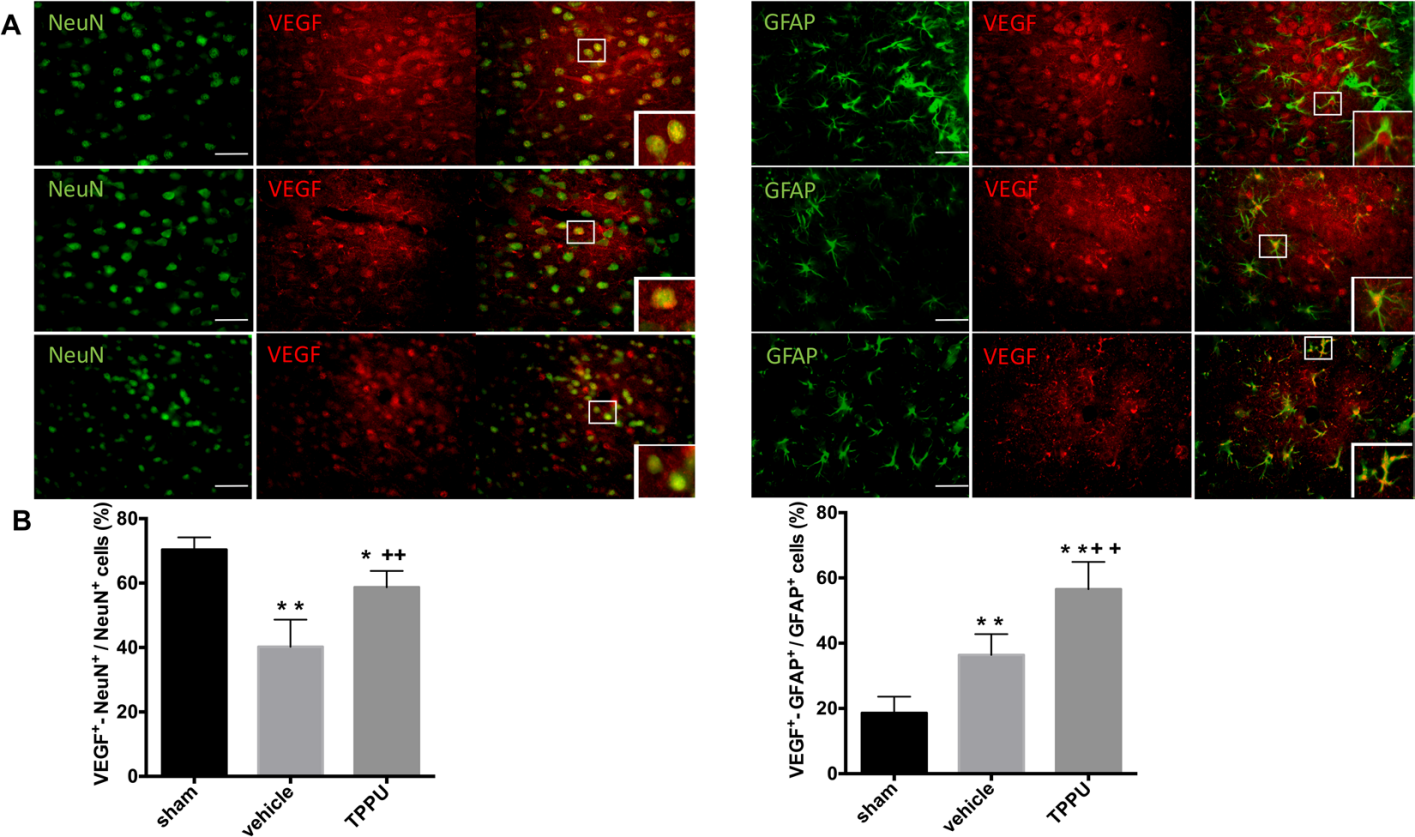
TPPU increases astrocyte-derived VEGF in the cortex at day 2 after MCAO and reperfusion. (**A**) Immunohistochemistry showed two main populations of VEGF-positive cells in ipsilateral cortex: neuron-like NeuN^+^-VEGF^+^ cells and astroglia-like GFAP^+^-VEGF^+^ cells. Scale bar = 50 μm for low power images. Area in white square is shown in inset at higher power. (**B**) Quantification of NeuN^+^-VEGF^+^ cells in the peri-ischemic cortex showed a decrease in the vehicle group that was attenuated by TPPU treatment. Vehicle-treated rats exhibited an increase in GFAP^+^-VEGF^+^ cells in the peri-ischemic cortex that was augmented by TPPU treatment. Groups were compared by ANOVA and the Holm-Sidak procedure for multiple comparisons (means ± SD; *n* = 5 rats per group; *p < 0.05, **p < 0.01 vs. sham group; ^++^*p* < 0.01 vs. vehicle group).

Previous work showed that application of TPPU to cultured astrocytes after oxygen-glucose deprivation increases VEGF expression and release^35^, which is consistent with the ability of EETs to promote VEGF release and tissue repair in a variety of organs^13^. Because VEGF promotes both neuronal protection and reparative mechanisms, we investigated the effect of TPPU on VEGF expression. We performed double immunostaining for VEGF with the NeuN or with the astrocyte marker GFAP on day 2 after MCAO (Fig. 8A,B). In the sham group, VEGF^+^-NeuN^+^ cells dotted abundantly in cerebral cortex. GFAP^+^ cells had small cell bodies with smooth processes, but only 19% of them double stained with VEGF. The percentage of VEGF^+^-NeuN^+^ cells decreased dramatically in the ipsilateral cortex after stroke, but TPPU rescued part of the loss (*p* < 0.001; *n* = 5 rats per group). As expected, GFAP^+^ cells displayed relatively larger cell bodies and thicker processes, typical of astrogliosis after ischemia. The percentage of VEGF^+^-GFAP^+^ cells increased significantly by 36% and 57% in the vehicle and TPPU groups, respectively, relative to that in the sham group. This increase was significantly greater in the TPPU group than in the vehicle group (*p* < 0.001). These data indicate a differential regulation of VEGF expression after MCAO between neurons and astrocytes and that TPPU treatment after reperfusion can increase the number of both neurons and astrocytes expressing VEGF.

## Discussion

In this study, we investigated whether administration of the sEH inhibitor TPPU at reperfusion after transient focal ischemia provides neuroprotective effects and explored possible mechanisms for this protection. The major findings of this study are that post-treatment with TPPU (1) suppresses brain sEH activity and significantly decreases infarct volume in both cortex and striatum, (2) significantly improves motor and sensory functions, (3) attenuates peri-infarct cortical neuron cell death, (4) effectively reduces expression of pro-inflammatory cytokines IL-1β and TNF-α and augments anti-inflammatory TGF-β during the acute phase of recovery, (5) decreases the number of peri-infarct microglia, and (6) promotes astrocyte expression of VEGF at 48 h after reperfusion.

Several studies of sEH inhibitors administered before or at the onset of MCAO in male mice and rats have confirmed a decrease in infarct volume, but without detectable changes in intraischemic perfusion, suggesting other mechanisms of action^8,21,22,24^. One study in mice reported a decrease in infarct volume when sEH inhibitor administration was delayed until reperfusion^8^. Our current finding that infarct volume was decreased when TPPU administration was delayed until reperfusion in rats is consistent with the concept that sEH inhibitors can act independently of effects on collateral blood flow during the period of MCAO. Moreover, our data extend previous reports by demonstrating that delaying sEH inhibition until after reperfusion can improve performance on a neurologic exam, the sticky tape sensory test, and a foot-fault sensorimotor test. Efficacy of sEH inhibitor administration at reperfusion may have clinical significance for adjunct therapy with thrombolysis and thrombectomy. However, a limitation of the present study is that we did not study aged animals or animals with cardiovascular co-morbidities. It will be important to test TPPU administration at reperfusion in aged rats of both sexes and with comorbidities for evaluating the full translation potential of this target.

Interestingly, we observed an increase in sEH expression and enzymatic activity after reperfusion. In non-ischemic brain, we observed sEH immunoreactivity in astrocytes and neurons, and others have also shown expression of sEH in blood vessels^16^. We did not explore whether the upregulation of sEH was limited to specific cell types, but others have recently reported enhanced sEH staining associated with astrogliosis after MCAO^23^. Our results show that the 1 mg/kg dosing of TPPU was sufficient to suppress the post-ischemic increase in brain sEH activity. We chose to use TPPU because it has high potency for inhibiting sEH, and a longer plasma half-life than many of the earlier generation of sEH inhibitors^32^. It also has greater water solubility, less binding to plasma proteins, and an extended resident time on the sEH molecule. Importantly for neuroprotection studies, it crosses an intact blood-brain barrier in rodents.

Based on these results, we investigated the factors that might contribute to the neuroprotective effects of post-ischemic TPPU administration. During the acute phase of ischemic stroke, microglia are activated rapidly, and inflammatory cytokines and chemokines are secreted largely by microglia and other cells to orchestrate the inflammatory response^36^. When we analyzed the expression of inflammatory factors, we found that TPPU administration decreased proinflammatory IL-1β and TNF-α expression but augmented anti-inflammatory TGF-β expression. This finding that an sEH inhibitor can limit expression of proinflammatory cytokines is consistent with a report showing decreased expression of inducible nitric oxide synthase after MCAO in rats administered an sEH inhibitor intraventricularly^23^. The latter finding suggests that the anti-inflammatory effect of an sEH inhibitor after MCAO can be attributed to actions within the brain and not necessarily by actions on the peripheral immune response. Also, consistent with a role for EETs in suppressing inflammation in brain after MCAO, overexpression of endothelial CYP enzymes that produce EETs limits the inflammatory response after MCAO^14^. After cardiac arrest, administration of an sEH inhibitor was reported to increase IL-10 induction in brain^30^. Unexpectedly, we failed to find an increase in IL-10 protein at 1 or 2 days of reperfusion with either vehicle or TPPU treatment in our focal stroke model. Perhaps longer recovery periods would be required to see an effect on this cytokine associated with infarct resolution and repair. Investigation of an extended panel of cytokines and chemokines at multiple time points is warranted in future studies to better characterize the effect of sEH inhibiton on the dynamics of the neuroinflammatory response.

An anti-inflammatory effect of TPPU is also supported by our observation that the normal increase in number of microglia in the peri-infarct cortex was attenuated by administration of TPPU. A similar attenuation has recently been reported by others with a different inhibitor^23^. It should be noted that the decrease in microglia number in our study was associated with relatively similar attenuation of TUNEL-positive neurons. Based on our data, we cannot distinguish whether TPPU’s effect on inflammation caused a decrease in neuronal cell death, or if a primary effect of TPPU on neuronal cell viability reduced the inflammatory response. Alternative mechanisms whereby TPPU exerts neuroprotection are by the inhibitory effect of EETs on reactive oxygen species generation, mitochondrial dysfunction^37^, and apoptotic signaling^5^ and by the vasodilatory effects of EETs^38,39^. A limitation of the present study is that we did not investigate oxidative stress, mitochondrial dysfunction, apoptotic signaling, or reperfusion defects in the microcirculation. In cell culture experiments, EETs and sEH inhibitors are capable of directly protecting primary cultured neurons and astrocytes from oxygen deprivation^5,22^, thereby indicating the potential for actions independent of immune cells and perfusion defects. Because of the low basal expression of sEH that we observed in microglia, reduced inflammation and the inhibition of microglial activation with the sEH inhibitor may be mediated indirectly by regulation of astrogliosis and attenuation of neuronal apoptosis^23^.

Our immunoreactivity results showed that sEH is well expressed in astrocytes. Astrocytes are the most abundant cells in brain, and one of their important functions is to release neurotrophic factors. VEGF is a trophic factor that protects neurons directly through Akt and ERK signaling pathways^40,41^. It also promotes angiogenesis^42^ and regeneration^13^. We immunostained sections of rat brain for the combination of VEGF and NeuN or GFAP. In the sham group, VEGF was predominantly associated with neurons and to a lesser extent with astrocytes. However, cell counts suggested that inhibition of sEH potently promoted astrocyte-derived VEGF in the cortical peri-infarct region. In the vehicle group, VEGF-positive neurons decreased markedly after MCAO and reperfusion. This decrease is likely due, in part, to the injury to neurons after ischemia and reperfusion. Furthermore, the increase in the number of VEGF-positive neurons after TPPU treatment may be secondary to the rescue of viable neurons, as supported by the decrease in TUNEL staining. Previous work in cell culture demonstrated that after oxygen-glucose deprivation, inhibition of sEH with TPPU dramatically increases astrocytic release of VEGF into the medium and that exposure of neurons to the astrocyte-conditioned medium rescued them from oxygen-glucose deprivation through VEGF receptor 2 signaling^35^. Another study reported that application of 14,15-EET directly to astrocytes after oxygen-glucose deprivation increases the release of brain-derived neurotrophic factor^43^. Therefore, one mechanism by which TPPU may exert protection after ischemia is through augmentation of trophic factor release from astrocytes. We speculate that the increase in trophic factor release by sEH inhibition could have a clinically relevant therapeutic window for brain repair and functional outcome.

In summary, our study provides support that targeting sEH specifically during the post-ischemic reperfusion period is beneficial. Daily administration of the potent sEH inhibitor TPPU reduced infarct volume and peri-infarct cell death, attenuated the neuroinflammatory response, promoted astrocytic VEGF expression, and improved performance of rats on sensorimotor tasks. Additional work is warranted to assess the therapeutic window for trophic factor release and the consequences for long-term functional outcome.

## Methods

### Surgical preparation

This study was approved by the Institutional Animal Care and Use Committee at the Johns Hopkins University, and all procedures on animals followed the approved protocol. Adult male Sprague-Dawley rats weighing 250–300 g were obtained from Charles River Laboratories (Frederick, MD). Anesthesia was induced in the rats with 5% isoflurane and maintained with 2% isoflurane during the surgery. MCAO was carried out with the intraluminal filament technique as previously described^44^. After the right common carotid artery was exposed and occluded through a lateral incision, the external carotid artery and the proximal pterygopalatine artery were ligated. A 4–0 nylon suture with a rounded tip was inserted into the internal carotid artery and maintained there for 90 min before reperfusion. At least a 60% reduction of blood flow was verified by laser-Doppler flowmetry. In rats that underwent sham surgery, the arteries were isolated but not ligated, and no filament was inserted. Temperature was maintained with a heating lamp at approximately 37 °C throughout the surgery and early reperfusion. Rats were randomly assigned to a sham surgery group or to treatment with either vehicle or TPPU after 90 min of MCAO. TPPU was completely dissolved in polyethylene glycol 400 and was administered by intraperitoneal injection in an aqueous solution of 10% (vol/vol) normal saline at a dose of 1 mg/kg^45^ at reperfusion and again 24 h later.

### Neurobehavioral tests

At 2 days of recovery, we performed a neurologic examination, a modified sticky tape test, and a foot-fault test. The neurologic examination was based on a modified neurological severity scoring protocol^46^ and is described in Supplementary Table S1. This examination provides an overall assessment of motor, sensory, balance, and reflex tests graded from 0 (normal score) to 18 (maximal severe score). The modified sticky tape test was performed as described^47^ and uses a non-removable tape sleeve wrapped around the forepaw The time that the rat spent attending to this stimulus was recorded over a 30-s observation period. Outcome performance was expressed as a ratio of left attention time/right attention time. For the foot-fault test, the rats were placed on a horizontal metal grid suspended over a mirror, which aided us in detecting foot slips during video playback. A foot-fault was defined as a forelimb or hindlimb missing or slipping off the grid. We counted the number of foot-faults for the limbs contralateral and ipsilateral to the ischemic injury as well as the total number of steps taken over a 5-min period^48^. The results were calculated as the percentage of contralateral limb foot-faults per limb step minus ipsilateral limb foot-faults per limb step. Investigators blinded to treatment group assessed neurobehavioral performance and evaluated the results.

### Infarct volume

At 48 h after reperfusion, the brain was harvested for measurement of infarct volume. Seven coronal sections (2-mm thick) were stained with a 1% solution of triphenyltetrazolium chloride (TTC)^44^. The infarcted area was traced and analyzed with Image J software (NIH, Bethesda, MD, USA) by a person blinded to treatment group.

### Western blotting

Brains were collected at 24 or 48 h after surgery for Western blotting. The tissues from the ipsilateral cortex were homogenized in RIPA buffer (Sigma-Aldrich, St. Louis, MO, USA) with protease inhibitor cocktail (ROCHE, Indianapolis, IN, USA) and then centrifuged at 10,000 *g* at 4 °C for 30 min. We collected the supernatants for protein quantification with the bicinchoninic acid assay. Equal amounts of each protein sample were separated by electrophoresis in 4–15% sodium dodecyl sulfate-polyacrylamide gels and transferred to polyvinylidene fluoride membranes. After being blocked, membranes were probed during overnight incubation at 4 °C with the following primary antibodies: mouse anti-β-actin (1:3000, sc-47778, Santa Cruz Biotechnology, Dallas, TX, USA), goat anti-sEH (1:50, sc-22344, Santa Cruz Biotechnology), rabbit anti-IL-1β (1:100, ab9722, Abcam, Cambridge, MA, USA), rabbit anti-IL-10 (1:500, ab9969, Abcam), and rabbit anti-TGF-β (1:500, ab66043, Abcam). We quantitatively analyzed the protein bands with Image J software to obtain the optical densities.

### Real-time PCR

We extracted total RNA from brain cortex with Trizol^®^ Reagent (Invitrogen, Karlsruhe, Germany) by following the manufacturer’s protocol. The first-strand cDNA was synthesized by High Capacity RNA-to-cDNA Kit (Applied Biosystems, Foster City, CA, USA). Quantitative real time-PCR was performed with Power SYBR@Green PCR Master Mix (Applied Biosystems) on an ABI 7500 FAST Real-Time PCR System (Applied Biosystems). The sequences of primers were as follows: forward 5′- CACCTCTCAAGCAGAGCACAG, reverse 5′-GGGTTCCATGGTGAAGTCAAC for IL-1 β; forward 5′-AGATGTGGAACTGGCAGAGG, reverse 5′-CCCATTTGGGAACTTCTCCT for TNF-α; forward 5′-CCTGCTCTTACTGGCTGGAG, reverse 5′-TGTCCAGCTGGTCCTTCTTT for IL-10; and forward 5′-AGATGTGGAACTGGCAGAGG, reverse 5′-CCCATTTGGGAACTTCTCCT for TGF-β. Expression levels of the target gene relative to glyceraldehyde-3-phosphate dehydrogenase (GAPDH) mRNA were determined with the ΔΔCT method.

### ELISA

Rat TNF-α levels in brain cortex tissue were measured by ELISA kits (TNF-α ELISA kit [B167329, Biolegend, San Diego, CA]) according to the manufacturers’ protocols. Activity of sEH in rat brain cortex was measured by incubating brain homogenate with 14,15-EET for 1 h and assaying for 14,15-DHET with an ELISA kit (DH1, Detroit R&D, Detroit, MI, USA). The optical density values were read on a plate reader (EL808, BioTek Instruments, Winooski, VT, USA) and normalized for protein concentration.

### Immunofluorescence

At 48 h after reperfusion, the rats were deeply anesthetized and perfused transcardially with phosphate-buffered saline (PBS) and 4% paraformaldehyde. The brains were fixed overnight in 4% paraformaldehyde and immersed in 30% sucrose. Frozen 25-μm coronal sections were immunostained. In brief, after being washed three times in PBS, sections were blocked by 5% serum for 1 h. The samples were then incubated with appropriate primary antibodies: GFAP (1:500, G3893, Sigma), NeuN (1:500, MAB377, Millipore, Billerica, MA, USA), Iba1 (1:500, 019–19741, Wako, Richmond, VA, USA), sEH (1:50, sc-22344, Santa Cruz Biotechnology), VEGF (1:200, ab46154, Abcam) and respective secondary antibodies (Alexa Fluor 488- or 594-tagged, 1:500, Invitrogen) in PBS with 5% serum. Images were acquired on a fluorescent microscope (200× ; Eclipse 90i, Nikon, Tokyo, Japan). Five sections per rat brain were analyzed blindly by counting immunopositive cells in five fields adjacent to the ischemic lesion in cortex per section (3 in dorsal cortex and 2 in ventral cortex) with the aid of Image J software (NIH Image, USA). For statistical analysis, the average value from 25 fields was used to obtain a single value for each rat.

### TUNEL Staining

Nonviable neuronal cells were detected by fluorescence with the *In Situ* Cell Death Detection Kit, POD (ROCHE, Indianapolis, IN, USA) according to the manufacturer’s instructions. Cells double stained with both NeuN and TUNEL were considered to be nonviable neurons. Their total number was obtained in a blinded fashion under light microscopy (200×; Eclipse 90i). Finally, an average for each group was calculated.

### Statistical Analysis

Data were analyzed by analysis of variance, and post hoc comparisons were made with the Holm-Sidak procedure. For real-time PCR data, we performed a logarithmic transformation to allow the data to pass the equal variance and normality tests. Because infarct volume, neurobehavioral data, and protein levels did not pass the normality test or equal variance test, we used nonparametric tests. For infarct volume, differences between the vehicle and TPPU groups were analyzed with the Mann-Whitney Rank Sum Test. For neurobehavioral data and protein-level data, the Kruskal-Wallis analysis of ranks test was used. When there was an overall significant effect, pairwise comparisons were made with the Mann-Whitney test. A significance level of 0.05 was used in all tests. All results were expressed as the mean ± SD or as medians, quartiles, and 95% intervals.

### Data Availability

Original data will be provided upon request of the corresponding author.

## Electronic supplementary material

Supplementary Information
