# Author Correction: Soluble epoxide hydrolase inhibition decreases reperfusion injury after focal cerebral ischemia

**DOI:** 10.1038/s41598-021-02023-6

**Published:** 2021-12-14

**Authors:** Ranran Tu, Jillian Armstrong, Kin Sing Stephen Lee, Bruce D. Hammock, Adam Sapirstein, Raymond C. Koehler

**Affiliations:** 1 Department of Neurology, Second Xiangya Hospital, Central South University, Changsha, China; 2 Department of Anesthesiology and Critical Care Medicine, Johns Hopkins University, Baltimore, MD USA; 3 Department of Entomology and Nematology and UCD Comprehensive Cancer Center, University of California, Davis, CA USA

Correction to: *Scientific Reports* 10.1038/s41598-018-23504-1, published online 27 March 2018

The original version of this Article contained errors in Figure 5.

In Figure 5, the image for the TPPU/NeuN panel was inadvertently duplicated for the sham condition. Therefore, the images presented for the sham/NeuN and sham/merge panels were incorrect. In addition, the sham brain included in Figure 5 was not representative, as it had a lower number of NeuN positive cells than the other brains.

The original Figure 5 and accompanying legend appears below.

**Figure 5:**
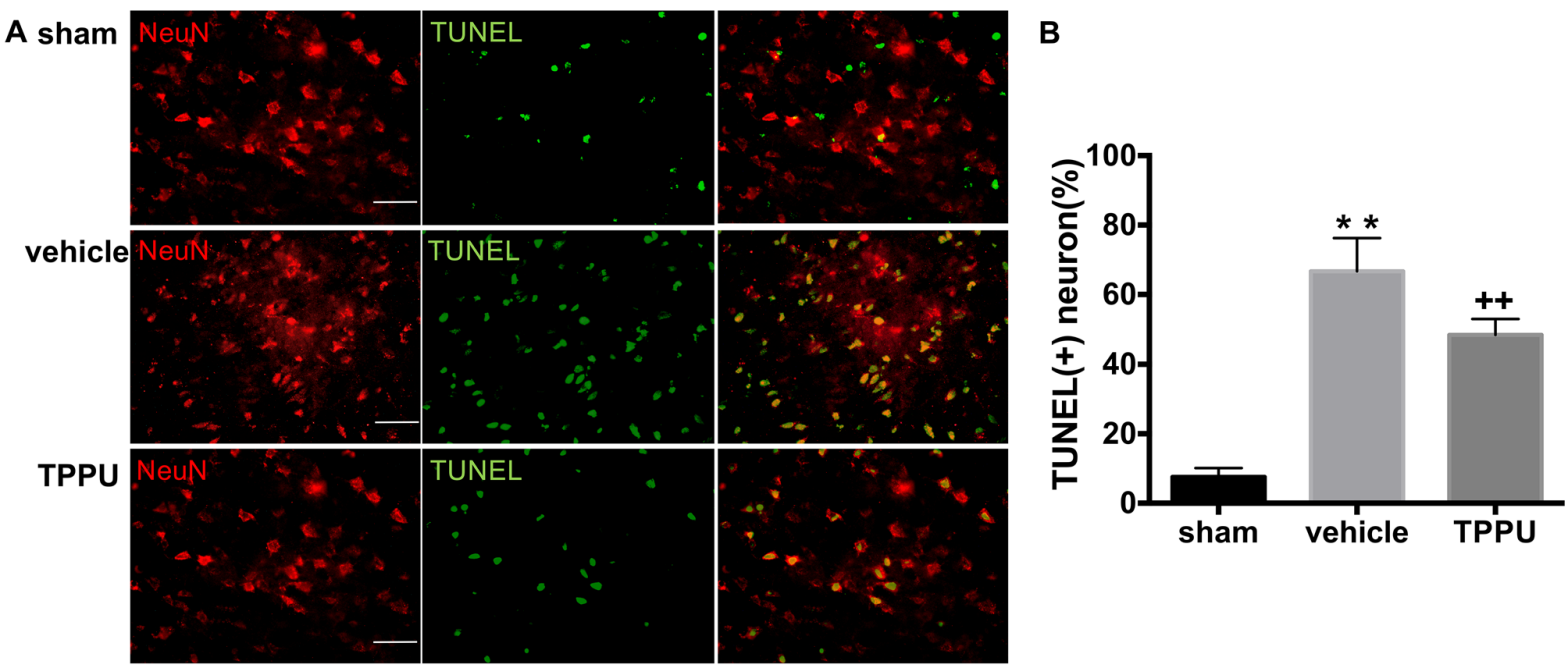
TPPU decreases peri-infarct neuronal cell death. Neuronal cell death in the ipsilateral cortex at 2 d after surgery was detected by terminal deoxynucleotidyl transferase dUTP nick end labeling (TUNEL, green) and NeuN (red) double staining. Scale bar = 50 μm. Sham, MCAO with vehicle, and MCAO with TPPU groups were compared by ANOVA and the Holm-Sidak procedure for multiple comparisons (means ± SD; *n* = 5 rats per group; **p < 0.01 vs. sham group, ^++^*p* < 0.01 vs. vehicle group).

The original Article has been corrected.

