# Supplementary material for: Soluble epoxide hydrolase inhibition decreases reperfusion injury after focal cerebral ischemia
Source: Sci Rep. 2018 Mar 27;8:5279. doi: 10.1038/s41598-018-23504-1 (PMC5869703; doi:10.1038/s41598-018-23504-1)

Soluble epoxide hydrolase inhibition decreases reperfusion injury after focal cerebral ischemia

Ranran Tu, Jillian Armstrong, Kin Sing Stephen Lee, Bruce D. Hammock, Adam Sapirstein, Raymond C. Koehler

Supplementary Information

Supplementary Table S1. Modified neurological severity score

Test	Possible points	Maximum points
Motor test: raising the rat by the tail		3
Flexion of forelimb	1	
Flexion of hind limb	1	
Head movement more than 10° to the vertical axis within 30 s	1	
Motor test: walking on the floor (normal=0; maximum=3)		3
Normal walk	0	
Inability to walk straight	1	
Circling toward the paretic side	2	
Falling down to the paretic side	3	
Sensory tests		2
Placing test (visual and tactile test)		
Proprioceptive test (deep sensation, pushing the paw against the table edge to stimulate limb muscles)		
Beam balance tests (normal=0; maximum=6)		6
Balances with steady posture	0	
Grasps side of beam	1	
Hugs the beam and one limb falls down from the beam	2	
Hugs the beam and two limbs fall down from the beam, or spins on the beam (>60 s)	3	
Attempts to balance on the beam but falls off (>40 s)	4	
Attempts to balance on the beam but falls off (>20 s)	5	
Falls off: no attempt to balance or hang on to the beam (<20 s)	6	
Reflexes absent and abnormal movements		4
Pinna reflex (a head shake when touched on the auditory meatus)	1	
Corneal reflex (an eye blink when lightly touched on the cornea with cotton)	1	
Startle reflex (a motor response to a brief noise from the snap of a clipboard)	1	
Seizures, myoclonus, myodystony	1	
Maximum points		18

One point is awarded for the inability to perform the tasks or for the lack of a tested reflex.
A score of 13–18 = severe injury, 7–12 = moderate injury, 1–6 = mild injury.

Supplementary Figure S1. Original gel of soluble epoxide hydrolase (sEH) and β-actin loading control, which was stripped from the same gel.

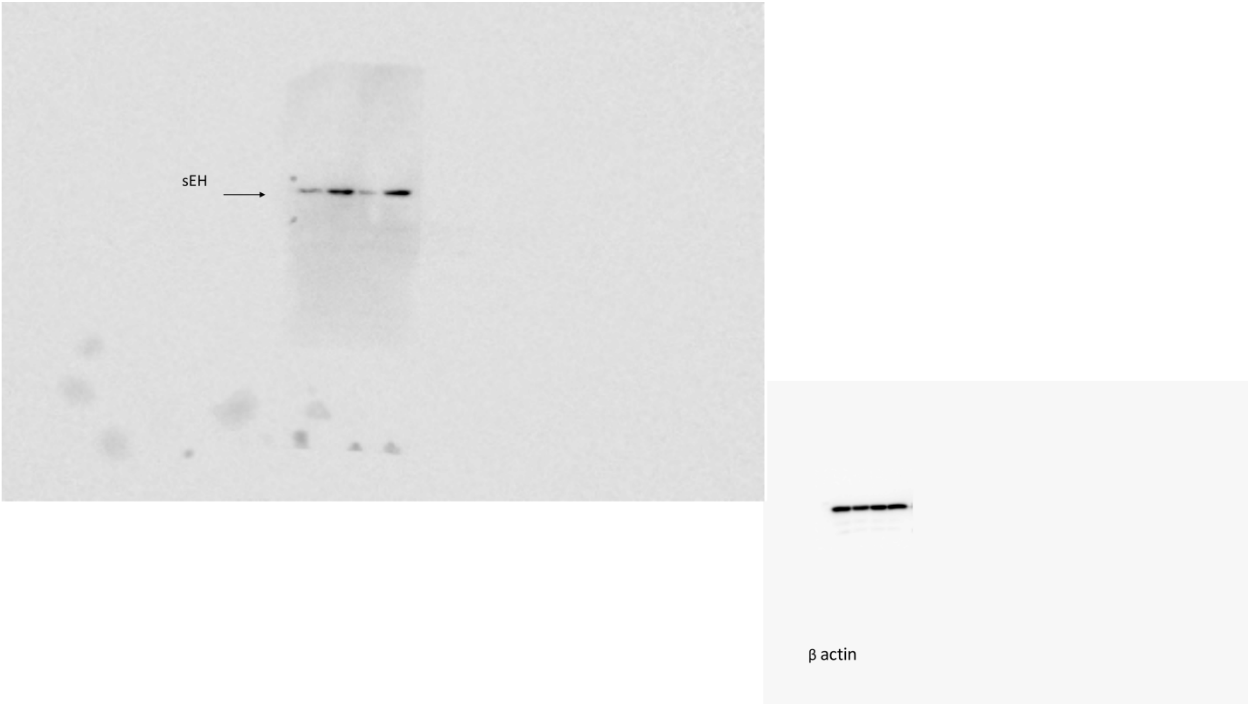

Supplementary Figure S2. Original gel of interleukin-10 (IL-10), IL-1b, TGF-b, and β-actin loading control. IL-10 was run on a separate gel, whereas others were stripped from the same gel.

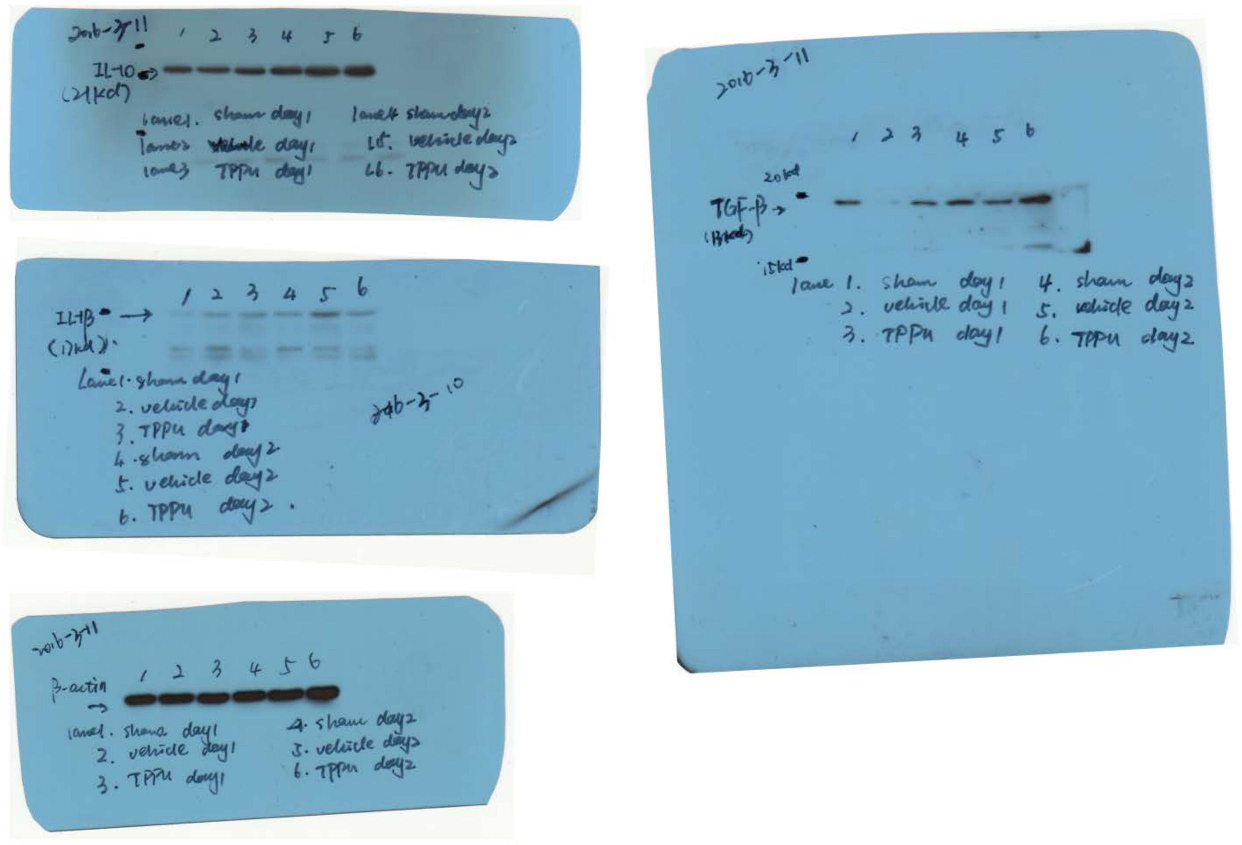

Supplement: Supplementary file 1 — Supplementary Information [file 41598_2018_23504_MOESM1_ESM.pdf]
